# Prodigiosin: a promising biomolecule with many potential biomedical applications

**DOI:** 10.1080/21655979.2022.2084498

**Published:** 2022-06-22

**Authors:** German A. Islan, Boris Rodenak-Kladniew, Nehuen Noacco, Nelson Duran, Guillermo R. Castro

**Affiliations:** a Desarrollo en Fermentaciones Industriales (CINDEFI), Facultad de Ciencias Exactas, Universidad Nacional de La Plata (UNLP) -CONICET (CCT La Plata)Laboratorio de Nanobiomateriales, Centro de Investigación y , La Plata, Argentina; b Facultad de Ciencias Médicas, Instituto de Investigaciones Bioquímicas de La Plata (INIBIOLP), CONICET-UNLP, CCT-La Plata, La Plata, Pcia de Bueos aires, Argentina; c Laboratory of Urogenital Carcinogenesis and Immunotherapy, Biological Institute, Department of Structural and Functional Biology, University of Campinas, Campinas, Brazil; d Nanomedicine Research Unit (Nanomed), Federal University of Abc (Ufabc), Santo André, Brazil; e. Partner Laboratory of the Max Planck Institute for Biophysical Chemistry (MPIbpC, MPG). Centro de Estudios Interdisciplinarios (CEI), Universidad Nacional de RosarioMax Planck Laboratory for Structural Biology, Chemistry and Molecular Biophysics of Rosario (MPLbioR, UNR-MPIbpC), Rosario, Argentina

**Keywords:** Prodigiosin, serratia marcescens, prodigiosin production, prodigiosin biological activities, anticancer, antimicrobial, antifungal

## Abstract

Pigments are among the most fascinating molecules found in nature and used by human civilizations since the prehistoric ages. Although most of the bio-dyes reported in the literature were discovered around the eighties, the necessity to explore novel compounds for new biological applications has made them resurface as potential alternatives. Prodigiosin (PG) is an alkaloid red bio-dye produced by diverse microorganisms and composed of a linear tripyrrole chemical structure. PG emerges as a really interesting tool since it shows a wide spectrum of biological activities, such as antibacterial, antifungal, algicidal, anti-Chagas, anti-amoebic, antimalarial, anticancer, antiparasitic, antiviral, and/or immunosuppressive. However, PG vehiculation into different delivery systems has been proposed since possesses low bioavailability because of its high hydrophobic character (XLogP3-AA = 4.5). In the present review, the general aspects of the PG correlated with synthesis, production process, and biological activities are reported. Besides, some of the most relevant PG delivery systems described in the literature, as well as novel unexplored applications to potentiate its biological activity in biomedical applications, are proposed.

## GRAPHICAL ABSTRACT

**Figure:**
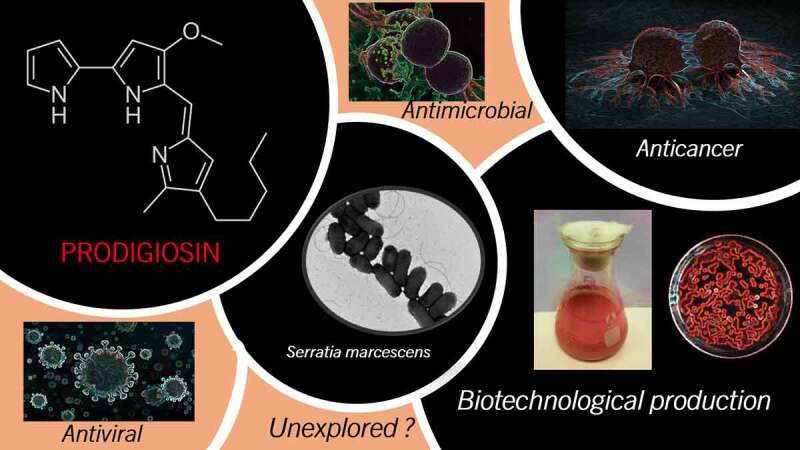

## Highlights

- Prodigiosin is a microbial alkaloid red pigment composed of a tripyrrole structure
- Prodigiosin displays antimicrobial, antifungal, and algicidal activities
- Cell disruption and extractive methods are required for prodigiosin purification
- Prodigiosin production can be modulated by environmental factors
- Prodigiosin chemical derivatives showed strong anticancer activities
- Encapsulation of prodigiosin displays enhanced biodisponibility

## 1. Introduction

In recent years, the use of natural pigments in different industrial areas, such as food, cosmetics, and health had a remarkable growth [1,2]. They appear as an alternative to replacing artificial synthetic colorants that are used to show hazardous side effects. Some of them had been removed from industrial applications due to evidence of carcinogenic effects and severe environmental issues [2]. On the other side, the potential antimicrobial activities of some bio-dyes become a promising alternative to deal with the increasing appearance of new antibiotic-resistant pathogens that represents a sanitary crisis and awakened the need to explore new molecules. Particularly, there are many advantages to the production of pigments by using microorganisms such as simple and fast growth in cheap culture media (*i.e*., agricultural, animal, and food wastes), short duplication time, high specific growth rate, simple purification process, biomass recovery, and weather independent production [3–5]. Also, bio-dyes could show some ‘extra’ potential biological activities, such as antioxidant, antiviral, antibacterial, and anticancer [6]. For example, violacein is a biomolecule synthesized principally by *Chromobacterium violaceum*, which possesses antiviral, antiprotozoal, anticancer, and antioxidant activities. Another compound is a derivative of the yellow-orange pigment flexirubin, ant342 (F-YOP), from *Flavobaterium sp*., that has been used for the treatment of tuberculosis [2,7].

Prodigiosin (PG) can be assigned to the group of bioactive colored molecules developed by microbial fermentation. PG is a red pigment mainly produced by *Serratia marcescens* strains and other microorganisms, that shows many promising potential therapeutic activities [8]. It was demonstrated to be an effective proapoptotic agent against multiple cancer cell lines including multi-drug resistance cells causing low or no toxicity on normal cell lines [9,10]. PG also displays antimicrobial, antiparasitic, insecticidal, and immunomodulatory activities [11,12]. Therefore, natural pigments like PG seem to be an attractive bioactive alternative and have been the subject of intensive research during the last decade.

Besides, the potentially beneficial applications of PG in the biomedical field, some limitations are obstructing the market entry. One of the main limitations is the main producer strain: *Serratia marcescens*, which is associated with some dangerous pathologies in mammalians [13]. Also, the lack of genetic information and metabolic networks of other PG-producer microorganisms is a serious limitation. Another point to be considered for industrial application is the lack of systematic production of PG in the laboratory and further scale-up using cheap substrates, mainly wastes. Additionally, the PG purification procedures reported requires the use of organic solvents for the extraction of the bio-dye from the cells which requires expensive and solvent-proof facilities at a large scale. Finally, the therapeutic application of PG demands systematic pre-clinical and clinical studies that were not performed at present, and also in one case were abandoned after phase clinical trials (*e.g*., obatoclax). Finally, PG paperwork was not sent to any national regulatory agency as far as we know, which is required for the development of therapeutic protocols, as well for compassionate use.

In the present review, the environmental factors affecting PG production as well as the major strategies for PG purification were described. The different biological activities and possible toxic effects were also discussed. As a novel point of study in comparison with other reviews about PG, the potential use of PG vehiculated into different delivery systems (nanoparticles, microcarriers, films, etc.) for applications in the biomedical field was reviewed. Finally, we proposed potential and unexplored applications of the pigment.

## 2. General properties of prodigiosin

The prodiginin family is a blood-red pigment group discovered in 1929, and a secondary metabolite produced by a large variety of microorganisms. The main prodiginine producers are Gram-negative bacteria, such as *Alteromonas rubra, Hahella chejuensis, Janthinobacterium lividum, Pseudoalteromonas denitrificans, Pseudomonas magneslorubra, Rugamonas rubra*, only a few species of *Serratia* (*i.e., S. marcescens, S. plymuthica*, and *S. rubidaea), Vibrio gazogenes, Vibrio psychroerythrous*, but also in members of the Gram-positive actinomycetes, such as like *Streptomyces coelicolor, Streptomyces fusant* NRCF69, *Streptomyces longisporusruber*, and *Streptoverticillium rubrireticuli, Zooshikela rubidus* [9,14–16].

**Figure 1.**
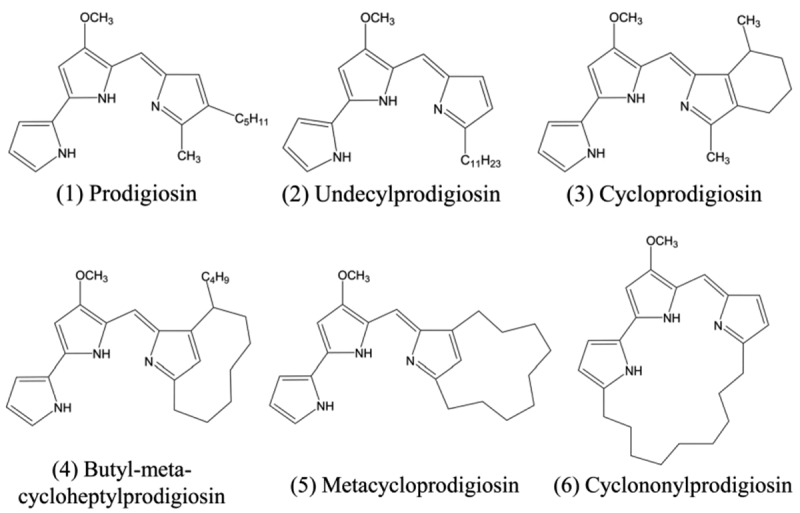
Comparative chemical structure of prodigiosin and its different isoforms (with permission from [18]).

From the chemical point of view, PG is a tripyrrole red pigment that contains a 4-methoxy- 2,2’-bipyrrole ring system directly connected with the third one by a methane bridge [17]. Different PG isoforms could be found in nature displaying different carbon chain lengths or molecular weights but still keeping the bioactive functions for medical and clinical applications (Figure 1) [18,19].

PG is a non-diffusible red pigment first characterized by *S. marcescens* and located in intracellular granules and extracellular and cell-associated vesicles but mainly attached to the inner membrane. Advanced spectroscopic and purification techniques revealed that a wide variety of compounds show a structure related to PG with the same pyrrolylpyrromethene core [20]. PG is synthesized by a bifurcated process, which ends with the enzymatic condensation of 2-methyl-3-n-amyl-pyrrole (MAP) and 4-methoxy-2,2ʹ-bipyrrole-5-carbaldehyde (MBC). The synthesis pathway is directed by the PG biosynthetic gene (pig) clusters, it begins with the transcription as a polycistronic mRNA from a promoter upstream of *pigA*. The genes *pigB-pigE* encode proteins for the biosynthesis of MAP and condensation with MBC producing PG and *pigF-pigN* encodes proteins that are related to the biosynthesis of MBC [4]. As a key enzyme in the PG biosynthesis pathway, *PigC* presents a bottleneck for efficient broad-range prodiginine production because of its limited substrate acceptance [21]. Gene complementation confirmed the regulatory function of the *EnvZ/OmpR* two-component regulatory system of genes *envZ* and *ompR* in PG production [22]. The final complex protein-PG can be traced at 500 nm, but the maximum pure PG can be observed at 535 nm and also determined by characteristic bands using RAMAN spectroscopy [23,24].

It is important to mention that PG is produced by an opportunistic pathogen. That means *S. marcescens* may cause infections in the urinary tract, eyes, skin, or lungs by direct contact with the bacteria. In the food industry, *S. marcescens* could cause food spoilage by contamination of milk or meat. The pathogenicity was associated with the biosynthesis of lipopolysaccharide, iron uptake, and the production of hemolysins [13]. However, a strategy to differentiate pathogenic from nonpathogenic strains is pigmentation. While environmental *S. marcescens* could be visualized as red colonies, the strains responsible for hospital outbreaks are mostly white (non-pigmented) [25]. Alternative to *S. marcescens*, a wide spectrum of natural Gram-negative and Gram-positive bacterial strains are capable of producing bio-dyes from the PG family with differences in the stimulating media and yield, or addition of functional groups to PG [26–28]. The first group of microorganisms (*S. marcescens, Serratia plymuthica, Serratia rubidaea, Hahella chejuensis, Pseudomonas magnesiorubra*, and *Vibrio psychroerythreus*) can synthesize the linear tripyrroles, including PG, with another sub-group (*Streptomyces longisporus ruber, Streptoverticillium rubrireticuli, Actinomadura madurae, Streptomyces coelicolor*, and *Saccharopolyspora sp*) that produces undecylprodigiosin. Other microorganisms belong to the macrocyclic group, characterized by ring formation between pyrrole A and pyrrole C, and mainly synthesize cyclononylprodigiosin (*Actinomadura pelletieri* and *Actinomadura madurae*). Finally, another group is known as the ‘cyclic group’ and produces two types of molecules that exhibit a ring on pyrrole C: cycloprodigiosin (*Vibrio gazogenes, Alteromonas rubra*, and *Pseudoalteromonas denitrificans*) and butylcycloheptyl prodiginine (*Saccharopolyspora sp* and *Streptomyces coelicolor*) [29].

The industrial production of PG by microbial fermentation represents an economical process, with a simple extraction and higher yields depending on strain selection and genetic improvement [9].

## 3. Environmental factors influencing Prodigiosin production

PG as a secondary metabolite appears only at the end of the exponential growth phase, while its production is influenced by the producer microorganism properties, its metabolic network, and diverse environmental factors, such as temperature, pH, ionic strength, and media composition including inorganic phosphate, and inorganic sulfate availability [3,30]. Also, it has been reported that the production of this pigment is limited by the negative feedback of product inhibition in batch cultures [31]. Besides, to the best of our knowledge, only one report recently described the production of prodigiosin in two-stage fermenters determined by Taguchi’s experimental statistical design. The results indicate that the prodigiosin production was increased up to 70 times [32]. Table 1 compiles the published works of PG production by mainly *S. marcescens*, but also from other members of the *Serratia* genus, such as *S. nematodiphila* [33,34] and the marine isolated *S. rubidaea* [35], but also other microbial sources, such as a member of the *Streptomyces* genus [36–38], *Achromobacter denitrificans* [39], and the marine isolates *Hahella chuejuensis* and *Zooshikella rubidus* [40]. Unfortunately, the information on genetic and metabolic pathways from microorganisms different from *S. marcescens* is scarce. The methodologies reported for PG production and scale of production, media, productivity (maximum PG concentrations and production time), as well as pH and temperature are also summarized in Table 1.

**Table 1.** Summary of PG production reported in the literature.

Strain	Methodology (volume)	Medium	Productivity (mg L^−1^ h^−^[^1]^) (Production/time)	Temperature (°C) – pH	References
*Serratia* spp. AM8887	Bioreactor (20 L)	6 g L^−1^ Sucrose, 6 g L^−1^, and glycerol, 15 g L^−1^ NaCl, and 12 g L^−1^ fertilizer waste	368.4 7,000 mg L^−1^/19 h	25°C – 6.5	[32]
*S. marcescens* WT	Bioreactor (7 L)	Saline medium	10.9 600 mg L^−1^/55 h	30°C – 6.0	[156]
*S. marcescens* UTM1	Bioreactor (5 L)	Brown sugar and lactose in nutrient broth	≈333.3 ≈8000 mg L^−1^/24 h	25°C – 7.0	[50]
*S. marcescens* NPLR1	Bioreactor (2 L)	Saline suppl. with tannery fleshing	1,200 48,000 mg L^−1^/40 h	30°C – 8.0	[157]
*S. marcescens* CF-53	Bioreactor (2 L)	Peanut oil cake extract	0.95 40 mg L^−1^/42 h	30°C – 7.0	[158]
*Serratia* sp. KH-95	Bioreactor coupled with extraction system (1 L)	20 g L^−1^ casein, 1 g L^−1^ MgSO_4_⋅7H2O, 1 g L^−1^ NaCl, and 1.7 g L^−1^ K_2_HPO_4_	400 4,800 mg L^−1^/12 h	28°C – 7.0	[159]
*S. marcescens* JX 1	Bioreactor (200 mL)	20 g L^−1^ glycerol, 2.0 g L^−1^ glycine, and 16.0 g L^−1^ Peptone	135.4 6,500 mg L^−1^/48 h	28°C – NR	[160]
*S. marcescens* WT	Bioreactor (100 mL)	1% M9 mineral suppl 1% Tannery fleshing	11.9 1,143 mg L^−1^/96th	30°C – 6.8	[161]
*S. marcescens* WT	Solid state fermentation (1 Kg)	Saline medium supplemented with wheat bran + Tannery fleshing	2,963.7 mg kg^−1^ h^−^[^1]^ 88,910 mg kg^−1^/30 days	29°C – 6.8	[162]
*S. marcescens* UCP 1549	Solid-state fermentation (5 g)	Saline medium suppl with food wastes: sugarcane bagasse, instant noodle waste, wheat bran, tangerine peels, pineapple peels, and crown	998.3 mg kg^−1^ h^−^[^1]^ 119,800 mg kg^−1^/120 h	28°C – NR	[163]
*S. marcescens* subsp. *lawsoniana*	Vessel (500 mL)	5 g L^−1^ maltose, 10 g L^−1^ peptone, 5 g L^−1^ NaCl, and 0.3 g L^−1^ sodium acetate	9.1 656 mg L^−1^/72 h	28°C – NR	[164]
*S. marcescens* MN5	Erlenmeyer (100 mL)	1% (v/v) of crude glycerol, or cotton seed cake, or soybean cake or 1% (w/v) black seed cake	333,3 48,000^#^/144	22°C – 9.0 Ranges of pH/temps	[51]
*S. marcescens* NITDPER1	Erlenmeyer (100 mL)	Peanut oil and palm oil broth	73.8–89.3 4,430–5,360 mg L^−1^/60 h	30°C – 7.0	[165]
*S. marcescens* SR_1_	Erlenmeyer (100 mL)	casein-enriched medium supplemented with 4% vegetable oil mixture (sunflower, coconut, and olive oil)	9.1 765.1 mg L^−1^/84 h	30°C – 7.0	[166]
*S. marcescens* ATCC 13880	Erlenmeyer (100 mL)	Sucrose and commercial or sheep wool peptones	2.43–4.74 175–341 mg L^−1^/72 h	25°C – 8.0	[167]
*S. marcescens* WT	Erlenmeyer (50 mL)	Powdered peanut seed broth	1,083 39,000 mg L^−1^/ 36 h	28°C – 7.0 Dif temps	[168]
*S. marcescens* WT	Erlenmeyer (50 mL)	Sesame and peanut seed broth	343.8–783.3 16,500–37,600 mg L^−1^ /48 h	28°C – 7.0 Dif temps	[169]
*S. marcescens* FZSF02	Erlenmeyer (50 mL)	Several combinations of carbon and nitrogen sources	4.9–214.2 353–15,420.9 mg L^−1^/72 h	26°C – 7.0	[170]
*S. marcescens* TKU011	Erlenmeyer (50 mL)	Saline suppl. with 1.50% squid pen powder/1.0% chitin plus 0.6% casein	103.3–192.5 2,480–4,620 mg L^−1^/24 h	25°C – 6.15 Ranges of pH/temps	[171]
*S. marcescens* TKU011	Erlenmeyer (50 mL)	Saline medium suppl with 0.5–2% powder of squid pen, shrimp head, crab shell, and shrimp shell	10.2 978 mg L^−1^/96 h	30/25°C – 7.0 Ranges of temps	[172]
*S. marcescens* SS-1 SMΔR*	Erlenmeyer (50 mL)	10 g L^−1^ tryptone, 5 g L^−1^ yeast extract, 2–6% vegetable oils	5.25–7.90 525–790 mg L^−1^/10 h	30°C – ≈9.0	[173]
*S. marcescens* MO-1	Erlenmeyer (50 mL)	1.00% mannitol, 0.40% yeast extract, and 0.40% ram horn peptone	5.8 278 mg L^−1^/48 h	28°C – 7.0	[174]
*S. marcescens* BWL1001	Erlenmeyer (25 mL)	Saline suppl. with 100 g L^−1^ soybean oil, 10 g L^−1^ peptone,	768.1 27,650 mg L^−1^/36 h	28°C – 5.0	[175]
*S. marcescens* S389	Sakaguchi flask (100 mL)	Saline medium suppl with 15% ethanol	41.7 3,000 mg L^−1^/72 h	28°C – 6.8	[176]
*S. marcescens* WT	Erlenmeyer (20 mL)	Proline, peptone plus Kitchen wastewater	20.7 870 mg L^−1^/42 h	28°C – 8.0	^[177]^
*S. marcescens* C3	Erlenmeyer immobilized cells on alginate (50 mL)	Saline suppl. with many Carbon and nitrogen sources	31.9–216.7 2,300 mg L^−1^ to 15,600 mg L^−1^/72 h	30°C – 6.0	[59]
*S. marcescens* WT	Erlenmeyer (NR)	Peanut seed broth, Sunflower seed broth/maltose, or glucose	31.8–33.2 1,525–1,595 mg L^−1^/48 h	28°C – 6.0	[178]
*S. marcescens* SK4-72	Erlenmeyer (NR)	2% Sucrose, 1.5%, beef extract and 0.75% L-proline	144.7 15,630 mg L^−1^/108 h	30°C – ≈9.0	[179]
*S. marcescens* WT	Erlenmeyer (NR)	Casein, *Madhuca latifolia*, powder, sweet potato, sesame, and nitrogen sources like casein, beef extract, urea	100 4,800 mg L^−^1/ 48 h	28°C – 7.0 Ranges of pH/temps IMP	[180]
*S. marcescens* JNB 5–1	Erlenmeyer (NR)	Synthetic medium	60.7 5,830 mg L^−1^/96 h	30°C – ≈9.0 Ranges of temps	[181]
*S. marcescens* JNB5-1	Erlenmeyer (NR)	2% Sucrose, 1.5%, beef extract and 0.75% L-proline	58.8 6,350 mg L^−1^/108 h	30°C – ≈9.0	[179]
*S. marcescens* BMJ 816 and AMJ 817	Erlenmeyer (NR)	1.60% Starch, 1.60% peptone and 1.0% trace compounds,	2.6 103 mg L^−1^/40 h	30°C – ≈9.0	[182]
*S. marcescens* UCP1549	Agar culture (20 mL)	2% mannitol medium suppl with 6% cassava waste	1,031 49,500 mg L^−1^/48 h	28°C – 7.0	[183]
*S. marcescens* ATCC 13880	Agar culture (20 mL)	Peanut seed, mustard seed, and sesame seed oil cakes nutrient agar	12.5 900 mg L^−1^/72 h	28°C – 7.5	[184]
*Serratia nematodiphila* YO1	Bioreactor (3.6 L)	Synthetic suppl. with vegetable oils	1.78–1.86 89.1–93.2 mg L^−1^/50 h	26°C – 7.0 Ranges of pH/temps	[34]
*Serratia nematodiphila* RL2	Erlenmeyer (50 mL)	Maltose, corn steep liquor, yeast extract	12.8 640 mg L^−1^/50 h	30°C – 6.0/7.0	[33]
*Streptomyces* sp. NRCF69*	Erlenmeyer (50 mL)	Saline medium suppl with 2.0% crushed peanut, sesame, or sunflower seeds, fenugreek, their oils, and coconut oil	391.7 47,000 mg L^−1^/120 h	28°C – 7.3	[36]
*Streptomyces* sp. JS520	Erlenmeyer (150 mL)	Maltose soy-yeast extract	0.96 138 mg L^−1^/144 h	30°C – 7.0 Ranges of pH/temps	[37]
Streptomyces coelicolor*	Agar plate	MS agar	0.90 96.8 mg g^−1^ DW/108 h	30°C – 7.2	[38]
*Achromobacter denitrificans* SP1	Test tube (10 mL)	Saline medium suppl. with Di (2-ethylhexyl) phthalate	7.3 x10^−^[^4]^^–^ 5.48 0.175–1,314 mg L^−1^/240 h	24°C – 8.8	[39]
*S. rubidaea* RAM_Alex	Erlenmeyer (25 mL)	5.0 g L^−1^ beef extract, 7.0 g L^−1^ peptone, 1.0 g L^−1^ yeast extract, 10.0 g L^−1^ NaCl	33.3 1,600 mg L^−1^/48 h	30°C – 6.0	[35]
*Hahella chuejuensis* KCTC2396	Agar plates	Marine broth 2216	0.59 28.10 mg L^−1^/48 h	30/37°C – 7.0	[40]
*Zooshikella rubidus* S1-1	Agar plates	Marine broth 2216	1.0 47.80 mg L^−1^/48 h	30/37°C – 7.0	[40]

Notes: NR, No Reported; WT, wild type; *, mutants; ^#^, estimated from Elkenawy et al., 2017 [51].

In general, high PG production at a laboratory scale was reported in semisynthetic media containing simple carbon sources, such as mono- or disaccharides such as fructose galactose, glucose, mannose, and sucrose [16]. However, there is evidence that PG production is modulated by glucose levels indirectly through cyclic AMP signaling, while gluconate inhibits pigmentation in *S. marcescens* through the *pig* cluster, more exactly *pigT* [41]. Another factor that can affect PG production is the presence of light since *S. marcescens* seems to produce more pigment in the absence of it [42,43]. Many studies proved that the presence of inorganic nitrogen, such as ammonium salts like ammonium sulfate, ammonium chloride, ammonium nitrate, and also urea inhibits PG production [44].

Many experimental conditions have been tested aiming for high pigment production at a laboratory scale, mostly based on nutrient broth and peptone-glycerol broth, and Luria Bertani (LB) modified media for *S. marcescens*.

The effect of Luria-Bertani (LB) broth and a modified LB (MLB) was also studied mainly for academic purposes, indicating that MLB presented almost 3 times more PG yield compared to the original. Furthermore, this MLB was supplemented with three inexpensive and easily available vegetable oils as carbon sources resulting in a marked enhancement of the pigment production: sunflower oil presented the best yield (790 mg L^−1^), approximately 14-fold higher than the obtained with LB broth, followed by olive oil (579 mg L^−1^) and soybean oil (525 mg L^−1^) [45]. It is interesting to realize that the average pH of LB medium is close to 9.0 [46]. These results suggested that PG can be synthesized between pH 5.0 and 9.0 mediated by the microbial strain metabolic pathway, media composition, and environmental frame (Table 1).

Other studies suggested that the addition of single amino acids induces the PG biosynthesis by alanine, histidine, and proline as the most effective ones [47]. As an example, *S. marcescens* isolated from irrigation water showed a maximum pigment production in nutrient broth supplemented with 10 mg ml^−1^ of L-tyrosine as a precursor amino acid inductor [48].

Two main aspects of PG production at a large scale that must be considered: high yields with low production costs, fermentation time, and sustainable production by rational use of natural resources compatible with environmental protection [49].

Recent sustainable approaches showed the bioconversion of agri-food industrial wastes as nutritional sources for the enhancement of microbial growth and PG production. For example, ram horn peptone (RHP) was used as an organic nitrogen source along with mannitol as a carbon source resulting in a PG yield of 278 mg L^−1^ in *S. marcescens*, probably because of the high presence of minerals and amino acids [44]. However, the productivity is very low, 5.8 mg L^−1^ h^−^[1], compared to other reports. In another work, *S. marcescens* was cultivated in a medium containing proline, peptone, and kitchen wastewater obtaining a productivity of 20.7 mg L^−1^ h^−^[1] which is 3.5 times higher than the previous one (see Table 1) [40]. In an interesting work, nutrient broth supplemented with brown sugar and lactose was studied for the production of PG by *S. marcescens* in a 5 L bioreactor showing 333,3 mg L^−1^ h^−^[1] PG productivity [50].

In recent work, the use of crude glycerol, a waste from biodiesel production, and cotton, black seed, or soybean cakes was employed for the production of PG at a laboratory scale with high success, the PG productivity was 333.3 mg L^−1^ h^−^[1] after 6 days cultivation [51]. Interestingly, biodegradation of the plasticizer Di (2-ethylhexyl) phthalate (DEHP), commonly used in many plastic objects, in the saline medium was explored at a laboratory scale to produce PG by *Achromobacter denitrificans* SP1. Besides the low PG productivity in the range of 7.3 x10^−^[4] to 5.48 mg L^−1^ h^−^[1], the approach was very remarkable since plastics are of high concern worldwide [39].

Similarly, *S. marcescens* ATCC 13888 cultivated medium supplemented with 8 g L^−1^ of sheep wool peptone as nitrogen source produced 341 mg L^−1^ PG compared with 302 mg L^−1^ and 175 mg L^−1^ of PG in media supplemented with commercial proteose peptone and tryptone peptone, respectively, but the productivity was low [52]. A different strategy for PG synthesis was made with fishery-processing wastes of squid pen powder (SPP) as both carbon and nitrogen (C/N) sources for *S. marcescens* achieving a high yield of PG (0.978 mg mL^−1^) using a medium with 1.5% SPP at 30°C for the development of maximum growth condition for 1 day and later at 25°C for 2 days to enhance PG production [53].

An increase in biomass growth and a better yield of PG were obtained with crushed sesame broth (16.68 mg mL^−1^). Also, recycled natural sources such as oil broths of coconut, peanut, and groundnut presented more pigment production than nutrient broth at 28–30°C [54]. For example, *S. marcescens* strain TUN02 cultured in a 14 L-fermenter with 4 L of groundnut oil of medium produced 6,89 g L^−1^ PG only in 4 h cultivation and without any commercial product [55].

A novel strain of *S. marcescens* UCP1459 isolated from soil produced a concentration of 49.5 g L^−1^ PG when cultured in 1.5% agar medium containing 6% ‘*manipueira*’ (cassava wastewater) supplemented with 2% mannitol at pH 7.0 and 28°C for 48 h. Also, *S. marcescens* UCP1459 produced pigments of varied colors, from yellow to dark red by changing the media composition from Corn steep to mannitol agar media [56]. A recent study of PG production by *S. marcescens* TNU01 in a 14 L-bioreactor containing 7 L of cassava wastewater medium supplemented with 0.25% casein, 0.05% magnesium sulfate, and 0.1% dibasic potassium phosphate at 28°C produced 6.15 g L^−1^ PG in for 8 h cultivation [57]. By comparing the two culture methodologies and assuming no extraordinary differences between both strains, it is clear that the production of PG in agar media is about 8-times superior to the liquid media under controlled biophysical parameters. However, agar media cannot be scaled up and is easy to develop in successive cultures just by recovering the cells and recycling using fed-batch and/or biomass recycling reactor techniques.

Another approach was to develop solid-state fermentations (SSF) using food wastes for the production of PG. In a recent report, the production of PG was screened at a laboratory scale using solid-state fermentation media containing individually or combined instant noodle waste, sugarcane bagasse, pineapple peels, crown tangerine peels, and wheat bran agro-industrial residues supplemented with saline solution and waste soybean oil. The fermentation was carried out at 28°C for 120 h and the maximum PG productivity was 998.3 mg kg^−1^ h^−^[1] substrate obtained with wheat bran residue [58]. In another SSF work, the PG productivity of a wild-type strain of *S. marcescens* was 2,963.7 mg kg^−1^ h^−^[1] in saline medium supplemented with wheat bran and tannery fleshing at a 1 kg scale (Arivizhivendhan *et al*., 2015).

An interesting strategy was developed for PG production using *S. marcescens* C3 cultivated in media containing starch and peptone at different carbon/nitrogen (C/N) ratios, and 30°C, pH = 6.0 for 72 h. The production of PG was increased from 2,300 mg L^−1^ to 7,050 mg L^−1^ by optimizing the C/N = 3/2. Later, *S. marcescens* C3 cells were immobilized on alginate gels, which results in 15,600 mg L^−1^ of PG [59].

The trend of PG synthesis and production showed that agro-industrial wastes can be used for large-scale production since they are cheap and, in some cases, pollutants, but also the PG productivity on average was higher than commercial and expensive media sources as displayed in Table 1. This conclusion was also supported by other microbial bio-dyes as previously reviewed [60,61].

Regarding the methodologies of PG production, the data in Table 1 reflect that in general SSF could be more useful since PG productivity is higher than liquid fermentation processes. Also, another advantage is the purification of the pigment since is located intracellularly and requires an extractive process using organic solvents.

Also, the physicochemical environmental parameters must be considered for PG production. In general, the selection of optimal pH, temperature ionic strength, and chemical media composition is related to the microbial strain (e.g., source of isolation, genetic information, metabolic network), and cultured methodologies (*e.g*., stirred tank, SSF, immobilized cells). Particularly, the conditions for microbial growth not necessarily are the best conditions for pigment production as previously reported [59]. As a typical example, *S. marcescens* can grow in the range of 5°C to 40°C, with an optimal temperature of 37°C. It was reported that high PG yields could be obtained in either minimal or complete medium after incubation at 27°C for 7 days. However, if the incubation temperature was raised over 37°C the PG production was stopped, which may be due to a decrease in the activity of a key enzyme responsible for PG synthesis [62].

Additionally, the temperature range for PG production is variable from 22°C to 30°C, similarly to the pH, ranging from 5.0 to approximately 9.0 in LB medium (Table 1). Additional information about the metabolic pathways and metabolism of the producer microorganisms are now challenged to improve the PG production on large scale.

## 4. Prodigiosin purification

The purification of PG is a critical step considering its diverse biological properties and immense potential in the pharmaceutical industry. For that reason, different processes to reach maximum PG amounts, a highly purified product, and a cost-effective extraction were proposed for commercial applications. Among the wide range of extraction methods, ultrasonication is possibly the most promising for scale-up and commercial application to obtain purified PG [63]. However, up till now, the efficiency of the different methods is variable, are not 100% effective, or implied the destruction of cells causing a loss of recycling steps and producing undesirable bacterial residual contaminations. Another challenge in the extraction/purification processes considering compounds from the PG family and other hydrophobic alkaloids is to diminish the use of large amounts of toxic and non-eco-friendly organic solvents (like chloroform, ethyl acetate, methanol, or others) [64].

**Figure 2.**
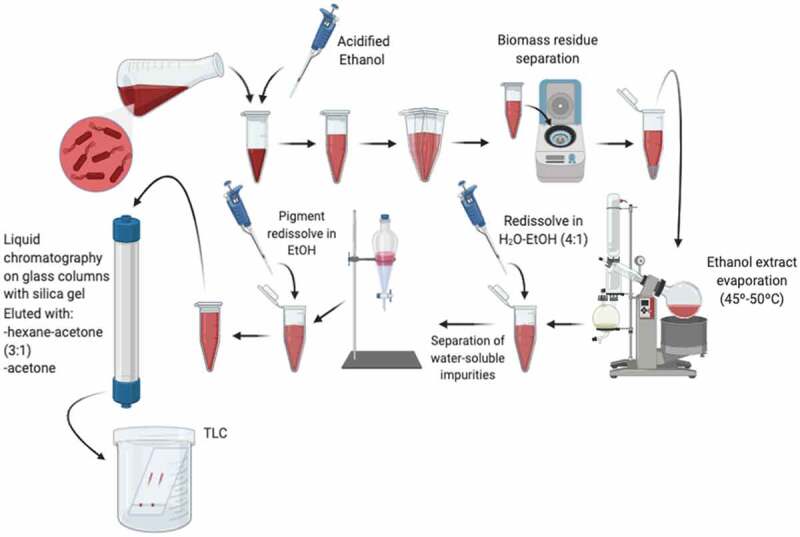
Schematic steps for Prodigiosin purification.

Typically, the PG purification process consists of a series of simple steps (Figure 2), some of which could vary according to different authors. Once fermentation is over, cells must be separated from the broth by centrifugation. Considering that PG is very hydrophobic (*e.g*., water-insoluble), the extraction generally consists of several steps involving organic solvents such as methanol, chloroform, hexane, and ethyl acetate, among others [31]. The pigment goes toward the organic phase during the extraction; hence the biomass starts to decolorate. In ethanolic extract evaporation proceeds at 45–50°C and the residue left behind can be redissolved with a water-ethanol mixture (4:1) for the separation of water-soluble impurities using a separating funnel. The pigment obtained is later dried in a vacuum dryer and resuspended in ethanol. The next step consists of liquid chromatography on a glass column with silica gel, in which this pigment resuspended generally in ethanol is applied to the sorbent and eluted with hexane-acetone (3:1) followed by acetone. Additionally, thin-layer chromatography (TLC) is done for further purification on glass silica gel 60 F254 plates using hexane-ethyl ether-acetic acid (70:30:1). The simple bands associated with PG are scraped off from the plates and eluted again from silica gel successively with a mixture of ethanol, acetone, and chloroform [9,15].

The purification process described above is not the only one, bibliographic research showed other purification strategies, such as extractive fermentation developed using a micellar aqueous two-phase system (ATPS) composed of a surfactant-rich phase (modified MGP medium with additional glycerol, double mineral concentration, and 2% of Triton X-114 or Tween 80 as extractive phase) for a batch fermentation [45,65]. Resulting in the production of 342 mg L^−1^ PG with a recovery of 81% in the extractive phase [66].

As was previously mentioned, six different extractions methods (homogenization, ultrasonication, freezing and thawing, heat treatment, organic solvents, and inorganic acids) were tested for PG evaluation yield. The results showed that ultrasonication presented the highest extraction yield among all the others with a 98.1 ± 1.7%, while freezing and thawing presented the lowest one (31.8 ± 3.8%). Besides, all these mentioned techniques are cell-destructive which avoids cell-recycling cultures and increases PG contamination with cell debris [63].

PG could be easily purified by running through a silica gel 60 columns with a suitable solvent system of n-hexane: toluene at the rate of 1:1 (v/v) combined with toluene: ethyl acetate at the rate of 9:1 (v/v) and gave 98% purity. A similar procedure was obtained in 92% purity [67,68].

## 5. Biological activities of Prodigiosin

One of the most relevant properties of PG is the wide spectrum of biological activities ranging from antimicrobial, anticancer, or antiviral to immunosuppressive activities that could be shown. Some of them are described in the present section as a reminder of how these activities can be related to new and unexplored drug delivery systems that could enhance the PG bioavailability.

### 5.1 Antimicrobial activity

The massive use of antibiotics has emerged the necessity to find novel drugs but also to control the environmental occurrence as well as the toxicity of new compounds [69]. In this sense, PG could provide not only new biological properties but also simple scale-up processes and reduced toxicity to the ecosystem and human health. Hence, the study of the inhibitory effect of this pigment suggests that it presents a better action against Gram-positive bacteria rather than Gram-negative. Studies that evaluated the antimicrobial activity of PG against pathogenic strains on disc-diffusion tests found significant inhibition zones for *Staphylococcus aureus* (35.0 ± 0.6 mm), *Enterococcus faecalis* (22.0 ± 1.0 mm), and *Streptococcus pyogenes* (14.0 ± 0.6 mm). Additionally, the minimum inhibitory concentrations (MIC) were determined for oxacillin-resistant *Staphylococcus aureus* (ORSA) and compared to oxacillin. *S. aureus* oxacillin-resistant strains presented higher sensitivity to PG with a lower MIC (lowest value, 1 μg mL^−1^) than the obtained for oxacillin alone (lowest value, 128 μg mL^−1^) [70]. Table 2 summarizes the antimicrobial spectrum of PG.

**Table 2.** Antibacterial activity of Prodigiosin.

Strain	Method	Concentration	Inhibition	Reference
*S. aureus*	Diffusion test	0.3 mg/disc	35.0 ± 0.6 mm	[70]
		0.15 mg/disc	60 mm	[71]
	MIC	5 µg/mL	–	[79]
	MBC	9 µg/mL	–	[73]
	Turbidity	0.18 µg/mL	30%	[11]
	CFU/mL	15% wt	97%	[144]
*E. coli*	Turbidity	100 µg/mL	30%	[11]
		0.64 mg/cm[^3]^	97.31%	[143]
		2 mg/mL*	55%	[135]
		120 µg/mL	40%	[185]
	Diffusion test	0.15 mg/disc	60 mm	[71]
	MIC	25 µg/mL	–	[79]
	MBC	20 µg/mL	–	[73]
*B. cereus*	Diffusion test	0.15 mg/disc	60 mm	[71]
	MBC	12 µg/mL	–	[73]
	Turbidity	0.64 mg/cm[^3]^	97.33%	[143]
		2 mg/mL*	40%	[135]
*P. aeruginosa*	MIC	15 µg/mL	–	[79]
	MBC	16 µg/mL	–	[73]
	CFU/mL	15% wt	98%	[144]
*E. fecaelis*	Diffusion test	0.3 mg/disc	22.0 ± 1.0 mm	[70]
*S. pyogenes*	Diffusion test	0.3 mg/disc	14.0 ± 0.6 mm	[70]
*C. botulinum*	Diffusion test	0.15 mg/disc	70 mm	[71]
*S. enterica*	Diffusion test	0.15 mg/disc	20 mm	[71]
*V. vulnificus*	Diffusion test	0.15 mg/disc	50 mm	[71]
*B. subtilis*	MIC	10 µg/mL	–	[79]
*S. thypi*	MIC	5 µg/mL	–	[79]

Antimicrobial activity of PG was also evaluated in agar plates by a diffusional technique using typical six foodborne pathogens: *Bacillus cereus* (MTCC 8372), *Clostridium botulinum* (ATCC 27022), *Escherichia coli* (MTCC 2939), *Salmonella enterica* (ATCC 29630), and *Vibrio vulnificus* (ATCC 27562). In most of the cases, the inhibition haloes were between 0.5 and 0.7 cm except for *V. vulnificus* which showed a 0.2 cm inhibition halo. Comparative analysis of bacterial growth inhibition of PG and sodium nitrite, a common food preservative, displayed similar growth inhibition results but about 25% advantageous for PG in *C. botulinum*. Later, an experiment using meat extract powder contaminated with bacteria showed a growth decrease of 7 log in presence of PG (6 x 10^1^ CFU g^−1^) compared to the control without any preserving agent (20 x 10^8^ CFU g^−1^) and better than sodium nitrite (32 x 10^6^ CFU g^−1^, 5 log difference). These results are indicatives of the potential application of PG in food compared with a traditional preservative [71].

Human pathogens such as *E. coli* and *S. aureus* were treated with PG aiming to study its effect and the primary sites targeted. After PG treatment, a 30% reduction in the growth rate for *E. coli*, and *S. aureus* was obtained at 100, and 0.18 μg mL^−1^, respectively, suggesting a high inhibitory activity. The treatment caused leakage of intracellular substances, especially in *S. aureus* which also presented signs of cell-surface damage. These results suggested that PG may act as a hydrophobic stressor being able to disrupt the plasma membrane *via* a chaotropic-mediated mode of action [11].

Besides, it was reported that oxacillin-resistant *E. coli* and *S. aureus* (ORSA), *Pseudomonas aeruginosa*, and *Acinetobacter spp*. resulted insensitive to PG. Meanwhile, PG showed antibacterial activity against *S. aureus, Staphylococcus saprophyticus, Enterococcus avium*, and *Streptococcus pyogenes* [70]. Also, this pigment seems to be a good curing agent on plasmids of *E. coli* HB101 and *S. aureus* [9]

The action mechanisms of PG are still poorly understood. Although some new data suggested that autolysis and other possible mechanism were occurring [11,72]. Besides, it was postulated based on the highly hydrophobic character of PG strongly interacting with cell membranes. A recent work developed using molecular dynamics established that PG goes at the interphase between cell membrane and water. The PG pyrrole rings interact within the lipid heads by hydrophobic interaction and with water *via* amine groups by hydrogen bridges. The PG covering the cell membrane surface interferes with the transport mechanisms, transmembrane translocation of lipids (i.e., flipflop mechanism), and solvation of proteins and transporters at the surface. Also, the increase of PG aggregates up to 8 molecules can be internalized inside the cell, but aggregates of higher than 8 PG molecules do not are capable to enter the cell membrane [73,74]. The authors hypothesized that PG interacts at the interface of the membrane and water through hydrophobic interactions and hydrogen bridges [74].

The molecular dynamic study agrees with previous work carried out in *E. coli* cells treated with high PG concentration showed more than 70% of Annexin V-positive, considered an inductor of apoptosis, which resulted in a bactericidal effect [73]. Moreover, despite there being no significant DNA damage or cytoplasmic membrane disintegration, the outer membrane was damaged after the treatment, and PG-treated cells presented morphological differences compared to control. Besides, the treated *E. coli* cells showed low levels of respiration activity, probably attributed to the PG binding, but also low protein and RNA synthesis and cell division were arrested [75]. These results are coincident with previous *in vitro* studies showing inhibition of topoisomerases type I and II by PG mediated by DNA intercalation [76].

Also, it was postulated a DNA gyrase (*i.e*., bacterial topoisomerase type II) and topoisomerase IV inhibition by PG using docking studies and the structural analogy with quinolones, which are cell growth inhibitors and interferes with main cellular mechanisms [77,78].

### 5.2 Antifungal activity

Different studies showed an interesting antifungal activity with a PG treatment (Table 3).

**Table 3.** Antifungal activity of Prodigiosin.

Strain	Method		Concentration	Inhibition (%)	Reference
*F. oxysporium*		MIC	8 µg mL^−1^	–	[79]
*A. flavus*		MIC	10 µg mL^−1^	–	[79]
*P. notatum*		MIC	22 µg mL^−1^	–	[79]
*Cladosporium*		MIC	20 µg mL^−1^	–	[79]
*C. albicans*		Turbidity	0.30 µg mL^−1^	30	[11]
*P. myriotylum*		Diffusion test	ND/crude extract	71.33	[80]
*R. solani*		Diffusion test	ND/crude extract	61.33	[80]
*S. rolfsii*		Diffusion test	ND/crude extract	49.33	[80]
*P. infestans*	Diffusion test		ND/crude extract	48.66	[80]
*F. oxysporum*	Diffusion test		ND/crude extract	31	[80]
*M. fijiensis*	Germ tube growth		996 µg mL^−1^	50	[81]
*B. cinerea*	Spore germination		10 µg mL^−1^	72	[82]

A susceptibility test made with pathogenic fungi (*Fusarium oxysporium, Aspergillus. flavus, Penicillium notatum, Claudosporium* spp.) showed low MIC in the range of 8–21 μg mL^−1^ compared to standard antifungal antibiotic nystatin [79].

In addition, PG presented a highly antifungal activity against phytopathogens, such as *Pythium myriotylum, Rhizoctonia s*olani, *Sclerotium rolfsii, Phytophthora infestans*, and *F. oxysporum* [80]. A similar toxic effect to that of benzimidazole was obtained when the PG was combined with chitinases, produced by *S. marcescens*, suggesting an interesting synergistic action against *Mycosphaerella fijiensis*, an ascomycete fungus responsible for black Sigatoka disease in banana [81]. This synergistic action was also observed against *Gray Mold Pathogen, Botrytis cinerea Persoon*. The inhibition of spore germination was tested with PG, endochitinase, or chitobiase, produced by *S. marcescens*. The germination rate was reduced by one-third when adding PG at 10 μg mL^−1^ or endochitinase, and one-sixth with the chitobiase treatment, compared to the control. The combination of the three compounds (PG at 1 μg mL^−1^) resulted in a synergic action achieving a one-third reduction in the germination rate [82].

### 5.3 Algicidal activity

The algicidal effects of PG have also been explored and some studies were directed to use this pigment as a biological bloom controller for harmful algae such as *Phaeocystis globosa*. Different concentrations of PG were added into *P. globosa* cultures and 5 μg mL^−1^ presented a better algicidal effect/dose-response reaching 84% algicidal activity in 72 h treatment. Also, PG showed an LD_50_ of 2.24 µg mL^−1^ in 24 h. Morphology effects were studied after PG treatment showing cell shrinkage and plasmolysis. Furthermore, as the exposure time increased, algal cells lysed and finally disintegrated. Additionally, normal cells that receive the treatment, lost their flagella and hence their mobility. These results indicated that PG presented good potential as an algicidal compound [83].

### 5.4 Prodigiosin and neglected diseases

There is wide experimental evidence of the potential use of PG against neglected diseases, such as Chagas disease, amebiasis, and malaria [84–86]. It was suggested that the mechanism by which PG may act is by triggering mitochondrial dysfunction. In one approach, a comparison between the action of PG and benznidazole (*i.e*., a drug currently used for the treatment of Chagas disease) was performed. Two different lineages of *Trypanosoma cruzi* were exposed to PG and both showed a lower IC_50_ compared to the IC_50_ of benznidazole. Additionally, the results of oxygen uptake and mitochondrial transmembrane potential assays, an anti-*T. cruzi* effect was elucidated, which consists of an apoptotic-like phenomenon induced by mitochondrial dysfunction caused by PG [87]. Also, effective inhibition of *T. b. gambiense* and *Plasmodium falciparum* was observed on PG compared to another bacterial pigment with similar potential uses (*i.e*., violacein) [88]. The authors suggest that mitochondrial inhibition may not be relevant given that bloodstream forms obtain ATP via glycolysis and do not express respiratory chain proteins. Instead, they suggest that PG could be inhibiting the cell cycle, or signal transduction, leading to apoptosis.

The anti-amebic potential of this pigment has been studied for a long time. The first record of a direct action of PG on *Entamoeba histolytica* was tested on two distinct strains, one associated with a mixed bacterial flora and the other one associated only with *Aerobacter aerogenes* presenting in both cases an *in vitro* cytotoxic effect [89]. In this study, the authors concluded that PG has a direct and strong effect on *E. histolystica*, discarding changes in oxidation/reduction potentials and pH associated with PG treatment. PG also presented cytotoxic activity on *E. histolytica* trophozoites resistant to metronidazole (MNZ), an antibiotic currently used for the treatment of amebiasis. Besides, a strong effect on mature cysts greater than MNZ was observed, suggesting that PG could be a therapeutic option in case of clinical MNZ resistance [90]. Natural occurring prodiginines showed potent *in vitro* activity against *P. falciparum* growth in the past, however, *in vivo* testing in mice has shown to extend survival but did not reach cure, or reached it but at concentrations above minimum toxic dose [91,92].

The antimalarial effect of heptyl PG in mice was studied against the *P. berghei* strain. Subcutaneous injections of this molecule in different concentrations gave a median survival time of 29 days compared to 7 days for the control. The increased survival seemed to be the result of a delay in the parasitemia during the first week. Nevertheless, no treatment was curative, and in all cases of surviving mice, they showed a rapid increase of parasitemia around the second week after infection [93]. In other research, several synthetic variations of prodiginines were tested and demonstrated that can be administered orally producing complete parasite clearance in mice [94]. However, some concerns associated with the toxicity of prodiginins suggest continuing to search for synthetic variants with reduced toxicity and improved antimalarial activity.

Another interesting study was carried out evaluating the PG mosquito larvicidal activity on *Aedes aegypti* and *Anopheles stephensi*. Crude extracted pigment was administrated studying the effect on different development stages of the mosquito. The LC_50_ and LC_90,_ expressed in µg mL^−1^, values obtained for the second, third, and fourth instars of *A. aegypti* were LC_50_ = 41.65, 139.51, 103.95; LC_90_ = 117.81, 213.68, 367.82 and for *A. stephensi* LC_50_ = 51.12, 105.52, 133.07; LC_90_ = 134.81, 204.45, 285.35, results showed that mortality rate increased with the increasing rate of dose. Time-dependent effects were studied as well, the pigment extract was highly larvicidal (maximum mortality >95%) at 500 ppm with 48 h of exposure against all tested instars of *A. aegypti* and *A. stephens*. That study was the first report on mosquito larvicidal activity of PG produced by *Serratia* species and presented a potential alternative to chemical pest control, taking into account that follow-up experiments against mosquitoes in the field are required until determining their full potential as alternatives to insecticides [95].

A similar study was carried out with *A. aegypti* and *A. stephensi* in presence of pure PG ranging from 3.9 to 62.5 µg ml^−1^. The results displayed 100% mortality for the II to IV larvae stages of *A. aegypti* and *A. stephensi* tested with the high PG concentration after 24 h incubation. Meanwhile, 90% and 84% mortalities for pupae stages of *A. aegypti* and *A. stephensi* under the same experimental conditions, respectively [96].

The study reveals severe changes in mosquitoes at all growth stages inhibited by PG. The major change can be attributed to the decrease of pH attributed to the inhibition of H^+^-ATPase pumps, also ascribed to dysfunction of the mitochondria in the gut [35]. In addition, changes in the activity enzyme patterns of acetylcholine esterase, proteases, and other esterases were observed as a product of PG treatments. As was mentioned in the previous paragraph, the first interactions of PG with cells are the lipid heads of and proteins of cellular membranes, which involve enzymes, transporters, and other molecules of the central metabolism.

### 5.5 Anti-cancer Activity

Numerous studies have been developed on the matter of PG and cancer treatments (Table 4). The primary hypothesis suggests that PG induces apoptosis in cancer cells, although it is not well understood the mechanisms by which this molecule acts. This has been observed in different human cancer cell lines in tissue culture, in hepatocellular carcinoma xenografts, and in human primary cancer cells [97].

**Table 4.** Anticancer activity of Prodigiosin.

Cancer type	Cell lines	Key findings	References
Gastric	HGT-1	− 4.0 µM PG decreased 80% cell viability, and induced DNA fragmentation and nuclear condensation	[186]
	BCRP EPG85- 257RDB	- IC50: 5–10 µM PG - PG is not an MDP protein substrate	[29]
Breast	KPL-1 MCF-7 MDA-MB-231 T-47D MKL-F	- IC50: 0-46 – 0.62 µM PG - 0.46 µM CycloPG induced G0/G1 cell cycle arrest and apoptosis in KPL-1 cells - Intracellular acidification was essential for the apoptosis	[97,187]
	MDA-MB-231 MDA-MB-468	- IC50: 0.06 − 0.26 µM PG (48 h) - PG induced caspase activation - PG suppressed MDA-MB-231 cell migration and invasion - PG inhibited tumor growth in an MDA-MB-231 xenograft mouse model - PG inhibited WNT/ β-Catenin signaling in breast cancer cells both *in vitro* and *in vivo*	[104]
	MCF-7 MDA-MB-231	- IC50: 1–2 µM PG - PG induced cyt C release, caspase activation, and PARP cleavage.	[98]
Lung	A549 (Dox-S) A549 (Dox-R)	- IC50: 10 µM for both cell lines - PG induced both autophagy and apoptosis	[103]
	GLC4	- IC50: 0.12–0.15 µM PG - PG induced cyt C release in cytosol	[99]
Colon	LD-1 c SW-620	− 1.1 µM PG induced DNA fragmentation and PARP cleavage (apoptotic markers)	[97,188]
Prostate	PC3	- PG (50 µg/mL) induced apoptosis through induction of p53 and Bax/Bcl-2 expression (mitochondrial pathway) - PG (100–250 µg/kg) inhibited tumor growth in PC3 tumor-bearing nude mice *in vivo*	[106]
Hematopoietic	Jurkat HL-60	- PG extract (10 µg/mL) induced apoptosis	[97,189]
Chronic lymphocytic leukemia	Patient-derived cells	− 0.1 µM PG inhibited 50% cell viability and promoted caspase activation	[97,190]
Neuroblastoma	SH-SY5Y LAN-1 IMR-32 SK-N-AS	- IC50: 0.7–7 µM PG - PG is a sequestering proton molecule that abolishes intracellular pH gradient, promoting ATP depletion - ATP depletion (starvation) leads to apoptosis	[100]

One of the most accepted anticancer mechanisms of PG includes mitochondrial dysfunction and ATP depletion [98–100]. Francisco *et al*. (2007) described PG as a proton sequestering agent that abolishes the intracellular pH gradient, therefore uncoupling the electronic chain transport of protons to mitochondrial ATP synthase, and promoting ATP depletion which leads to apoptotic cell death in neuroblastoma cancer cells [100]. Moreover, Llagostera *et al.*, (2003) demonstrated that PG induced apoptosis in small cell lung cancer cells via the mitochondrial pathway, promoting cytochrome c release [99].

Another interesting result was obtained by testing PG against estrogen receptor-positive (MCF-7) and negative (MDA-MB-231) breast cancer cell lines and the multidrug-resistant (MDR) MCF-7 cells. In this case, PG seemed to act promoting apoptosis via the mitochondrial pathway, causing dose- and time-dependent cytotoxicity on the three cell lines. Although MCF-7 cells were less affected by this pigment, it was discarded the fact that PG may be a substrate for MDR transporter molecules [98].

Some studies suggested that PG could promote double-strand DNA cleavage leading to apoptosis. Also, it had been seen that this pigment promoted H^+^/Cl^−^ symport by decoupling vacuolar H^+^-ATPase as PG binds Cl^−^ electrostatically promoting proton-coupled transmembrane transport of halides. This ability suggested that PG could be a useful pH regulator for targeting tumors with intracellular acidity increased [101].

The apoptotic action of PG was tested on a human gastric carcinoma cell line (HGT-l) showing a constant decrease in cell viability mediated by apoptosis. There was also morphological evidence, such as cell shrinkage, chromatin condensation, and more, suggesting PG as an apoptotic agent [102].

PG treatment was studied against doxorubicin-sensitive (Dox-S) and doxorubicin-resistant (Dox-R) lung cancer cells *in vitro* and *in vivo*. Similar results were obtained for cytotoxicity on both cell lines with a 10 μM IC_50_. The cell death of both cell lines was categorized as autophagy but also subpopulations of cells presented apoptotic features. Also, after PG treatment, tumors located in the mice trachea were attenuated indicating an effective action of PG against both Dox-S and Dox-R lung cancer [103]. Induction of apoptosis by PG in breast cancer cells in cell culture as well as in the *in vivo* experiments was observed. The results suggested that PG may inhibit cell growth, migration, and invasion by blocking Wnt/β-catenin signaling, which plays an important role in the development and progression of breast cancer [104].

PG was tested on both multidrug-resistant human gastric and ovarian cancer cell lines (overexpressing MDR1, BCRP, or MRP2 pumps) and their non-MDR type. It was found that this pigment produced a nearly identical cytotoxicity effect to the one on the parental lines, with no significant differences compared to cisplatin, daunorubicin, and mitoxantrone. Also, a FACS analysis showed that, unlike daunorubicin or mitoxantrone that could be effectively exported by the ABC pumps, PG could not be taken out of the human gastric carcinoma and human epithelial ovarian cancer cells [29]. Those results were suggesting that PG was not acting as an MDR protein substrate and for that reason could be a potential tool for the treatment of cancer cells that overexpress MDR transporters. In other work, purified PG obtained from the fermentation of marine chitins demonstrated potent anticancer activities against A549, HepG2, MCF-7, and WiDr cells with IC_50_ =_ _0.06, 0.04, 0.04, and 0.2 µg mL^−1^, respectively. The reported IC_50_ was 2.75-fold, 1.67-fold, and 3.25-fold more efficient than Mitomycin C, a well-known anti-cancer drug, against MCF-7, A549, and Hep G2, respectively [105]. In another study, PG was purified and tested on human choriocarcinoma (JEG3) and prostate cancer cell lines (PC3). In the *in vitro* assay, PG produced a dose-dependent apoptotic effect on JEG3 cells. Furthermore, PG presented interesting results in the *in vivo* assay, since the growth of JEG3 and PC3 tumors was considerably inhibited with dose and time dependence. It seemed that this molecule triggered an apoptotic response mainly through the mitochondria-cytochrome c pathway due to the induction of caspase-3 and caspase-9 activation, and the subsequent proteolysis of poly(ADP-ribose) polymerase (PARP) [106]. A similar way of action was observed when two small cell lung carcinoma (SCLC) cell lines, GLC4 and its derived doxorubicin-resistant GLC4/ADR cell line, were treated with PG, apoptosis was induced by cytochrome C release, caspase cascade activation, and PARP cleavage, in a dose–response manner. Additionally, it could overcome the multidrug resistance phenotype as it showed no difference between the two cell lines [107].

The combination of PG with a promising potent water-soluble purine-analog, PU-H71, which presents a higher affinity toward tumorigenic cells and is being tested in phase I clinical trials, was studied using different doses against triple-negative breast cancer MDA-MB-231 cells. The combination of half of the IC_50_ of both components (PG = 2.1 µM and PU-H71 = 157.88 nM) presented the maximum inhibition (75.14%) compared to other combinations. Regarding morphological changes, the effect of the combination of these components showed an increase in the number of floating cells and a more spherical appearance, related to higher cytotoxicity compared to untreated and DMSO-treated cells; suggesting a synergistic effect, which was demonstrated by the CI method (CI = 0.7). This synergistic action between both components was observed as caspase 3, 8, and 9 levels were increased (related to the apoptotic action of PG) and a remarkable decrease of HSP90α (Heat shock protein 90, a small family of chaperones considered as the key regulator of proteostasis) transcription and expression levels (related to PU-H71 action) [108].

**Figure 3.**
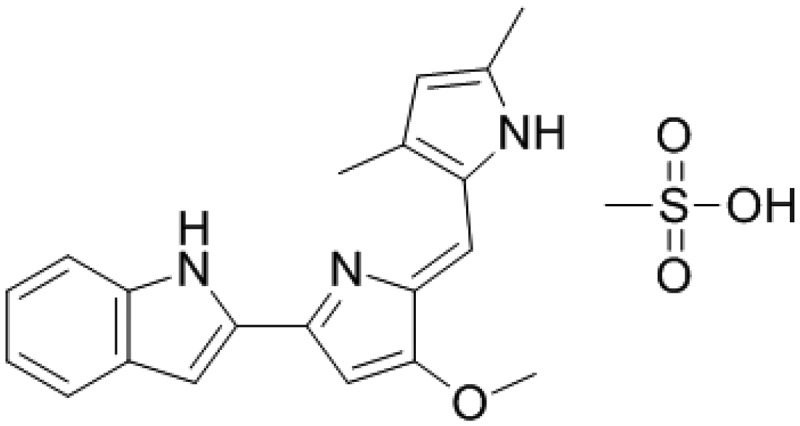
Obatoclax mesylate, a derivative of prodigiosin with high anticancer activity.

Also, a synthetic derivative of PG, commercially named Obatoclax mesylate or GX15-070 (CAS 803712–79-0) showed antineoplastic and pro-apoptotic properties (Figure 3). Obatoclax is a pan-antagonist of the BCL-2 protein family (*i.e*., the BCL-2, -xl, and -w plus Mcl-1) which are over-expressed in numerous cancer types. Obatoclax was tested alone and combined with many other anticancer drugs in phase I and II clinical trials for the treatment of several types of cancer including leukemias and lymphomas with promissory results [109,110].

However, phase III of clinical trials of obatoclax for lung cancer was abandoned. The main arguments to halt phase III were thrombocytopenia and ataxia at toxic levels. Besides, all cancer drugs available in the market display high toxicity [111,112]. However, the poly(lactic-co-glycolic acid) (PLGA) was used and combined with the red-blood-cell membrane (RBCm) to demonstrate that exerted stronger antitumor efficacy *in vivo* against lung cancer progression with superior safety [113]. Alternatively, the low solubility of obatoclax under physiological conditions (XlogP3-AA = 3.3) represented a serious issue for intravenous administration to the patients a decade ago. However, the developments of novel systems based on today’s nanotechnological tools can provide several advantages such as improving the biological activities of anticancer drugs, targeted nanoparticles with specific markers to tumor cells, plus a reduced amount of doses and increased patient compliance, etc. [114].

Based on the experimental experience accumulated for the treatment of cancers with obatoclax, several molecules derived from obatoclax were chemically synthesized, characterized, and *in vitro* tested as anticancer agents for the creation of a new anticancer arsenal [115].

PG inhibited cholangiocarcinoma (CCA) cell proliferation via suppressing SNAREs-dependent autophagy, indicating that PG could be a potential chemotherapy drug for advanced CCA [116].

Treatment with 30–100 ng/ml PG for 4–7 h decreased viability in THP-1 cells. In contrast to cancerous cells treated with PG, the non-cancerous kidney epithelial Vero cell line was not susceptible to various doses of PG [16].

PG showed anti-tumor activity *in vivo* in Lewis lung carcinoma-induced tumors in BALB/c mice. The tumor volumes in PG treated groups decreased by 34.18% after 28 days of administration [67].

### 5.6 Anti-viral activity

The antiviral effects of PG have been poorly explored. However, given that PG has been shown to interfere with signal transduction pathways, which are also essential for viral infection [117,118], there is an increasing interest to evaluate the potential of PG as an antiviral agent.

In a study performed *in silico* through homology modeling and docking analysis [119], the activity of PG against a variety of target proteins from viruses, such as the hepatitis B virus (HBV), human immunodeficiency virus (HIV), hepatitis C virus (HCV), and influenza A virus (H1N1) was analyzed. They found that PG has a promising antiviral potential against HBV, HIV, and H1N1 but not for HCV. However, these findings need to be confirmed *in vitro* and/or *in vivo* experiments.

The first study revealing the virucidal properties of PG was reported by Zhou and collaborators [120]. The authors explored the ability of PG to inhibit *Bombyx mori* nucleopolyhedrovirus (*BmNPV)* infection in silkworm *BmN* cells, as an *in vitro* cell model expected to be valuable for screening antiviral agents for human DNA virus. They found that PG at non-cytotoxic levels for uninfected cells (300 nM) resulted in strongly toxic to BmN cells. Indeed, PG at concentrations from 10 nM inhibited occlusion bodies (OB) formation, which represents the final and lytic stage of *BmNPV*, and budded virus (BV) production. Time- and dose-dependent experiments performed suggest that PG had both antiviral effects against *BmNPV* and selective cytotoxicity for *BmNPV*-infected cells depending on concentrations employed (10–300 nM). The antiviral mechanisms of action of PG in *BmN* cells include inhibition of viral DNA replication and gene transcription of early (*ie-1*), late (*vp39*), and very late (*p10*) genes involved in DNA replication, capsid formation, and OB production, respectively. Gene expression of *ie-1, vp39*, and *p10* was suppressed 55 to 100-fold after exposing cells to 100 nM PG for up to 72 h, suggesting that PG significantly inhibited viral gene transcription, and therefore, reduced both BV and OB production.

Another interesting study recently performed by Suryawanshi *et al*. evaluated the antiviral activity of PG against *Herpes simplex virus* (HSV) 1 and 2 [121]. It was observed that 2.5 µM PG strongly inhibited HSV-1 infection in human corneal epithelial (HCE) cells, a physiologically relevant *in vitro* model. The viral replication, transcription, protein synthesis, and egress of infectious virus particles were inhibited from concentrations as low as 0.3 µM PG.

PG was also able to suppress corneal HSV-1 infection *in vivo* in a mouse model. BALB/c mice infected with HSV-1 strain were topically treated with 50 µM PG, 50 µM Trifluorothymidine (TFT, a standard drug for ocular HSV infection), or DMSO (control), applied every 2 days for 7 days. PG significantly protected mice from the development of disease at levels similar to TFT. In addition, the prophylactic activity of PG against HSV infection was evaluated in HCE cells. The authors concluded that PG induced a prophylactic activity but did not interfere with viral entry, proposing that PG targets host signaling pathways once the virus infected the cells. More specifically, they show that PG exerts its antiviral activity through the inhibition of NF-κB and Akt signaling, which is involved in host cell survival and apoptosis inhibition and is activated during HSV infection.

In other work, a derivative of PG named obatoclax showed antiviral activity against alphaviruses such as Chikungunya (CHIKV) and Semliki Forest (SFV) viruses [122]. The mechanism of obatoclax was postulated as the inhibition of viral fusion to cells mediated by the naturalization of endosomal pH. Further antiviral research on SARS-Cov-2 with obatoclax showed inhibition of replication in epithelial cell cultures, and the mechanism of inhibition was attributed also to the changes in the endosomal acidification, and the impairment of furin and cathepsin activities, both enzymes involved in the activation of the viral fusion protein [123,124].

PG *in vitro* and *ex vivo* studies demonstrated inhibition of HSV-1 virus replication in a dose-dependent manner. Action *in vivo* ocular infection of HSV-1 showed a protective against disease development. Histopathology analysis demonstrated no inflammation of epithelial layers and conserved the corneal integrity. The mechanistic analyses showed inhibition of proviral host factors including NFkB/protein kinase B (AKT) in the PG group. These facts showed that PG acted by blocking the dysregulation of multiple signaling pathways during infection, and also interacted as host machinery to reduce viral replication and virus progression [125].

## 6. Toxicity effect of prodigiosin

Despite all the attractive potentials this molecule presents, it is strongly desirable to study the possible side effects or toxicity that it could generate before introducing it as a new therapy, either alone or as a complementary compound for an already used drug. Scarce literature is available about the toxicity of PG.

Pure PG was tested *in vivo* to evaluate its genotoxic potential on erythrocytes of peripheral blood of mice by microscopic registration of cells with micronuclei (PCE). Three PG concentrations (25.0, 8.0, and 4.0 µg kg^−1^) were injected subcutaneously into mice, after 48 and 72 h blood samples were taken and analyzed by light microscopy. The results showed an insignificant induction of micronuclei in PCE at all concentrations, an effect not dependent on dose, suggesting that PG did not produce a genotoxic effect [126]. The immunosuppressive effect of PG was studied showing inhibition of T-cell immune response at nontoxic concentrations. This effect was comparable to other T-cell-specific immunosuppressants, such as cyclosporin A and FK-506. Also, B-cell activity such as activated polyclonal antibody production was not affected at the same range of concentrations.

*In vitro* and *in vivo* assays were performed in which lymphocytes were cultured in the presence of PG and incubated for 5 days showing no toxicity at effective concentrations (PG < 100 nM). For the *in vivo* assay, B5C2F0 mice were injected with PG intraperitoneally at doses of 10 and 30 mg kg^−1^ daily for 3 days. The spleen was analyzed for the determination of antibody production by the PFC assay. The results showed that PG did not produce a toxic effect at both dosages [127].

Based on the potential trypanocide activity of PG, it was tested on the V79 fibroblast cell line and compared to Benznidazole, a trypanocidal drug. Three independent assays were done to evaluate cytotoxicity: the amount of nucleic acid content (NAC), MTT reduction, and neutral red uptake (NRU). PG showed IC50 values of 1–20 µM on the three tests, while benznidazole presented lower toxicity with IC_50_ of 2.0 mM. Nevertheless, due to its trypanocidal activity being 30-fold higher than that of Nifurtimox, a known trypanocide, PG may continue to be studied [85].

Treatment with PG exhibited no oral toxicity in mice. PG altered the cecum microbiota fullness and variety; interesting that the relative abundance of *Desulfovibrio* spp. substantially diminished, while *Lactobacillus reuteri* significantly augmented. Then, oral administration of PG has a beneficial effect on the intestinal microbiota of mice and suggests that it is a promising drug for intestinal inflammation therapy [128].

## 7. Prodigiosin and Drug Delivery

As was previously described, PG exhibits plenty of potential activities and some research has been made to enhance these properties or promote synergistic action with other bioactive compounds to develop new treatments or improve current ones. The challenges in implementing PG in drug delivery systems lie in achieving an increase in the bioavailability of the drug, considering that PG exhibits high hydrophobicity (XLogP3-AA = 4.5), which limits its application and effectivity in physiological environments. In this sense, the different vehicles could provide an improvement in three factors: dissolution, permeability, and solubility of PG. Additionally, nanocarrier could overcome biological barriers, decrease the PG toxicity in normal cells, provide a controlled therapeutic release of PG, and selectively target PG to specific regions of the body to be treated [129].

There are a wide variety of materials that have been used for drug delivery research, including biopolymers, metal particles, lipids, and others. In Table 5 there is a summary of all the systems described below and their potential use.

**Table 5.** Different drug delivery systems for prodigiosin vehiculation.

Delivery system	Design	EE (%)/DL*	Application	References
*PG loaded Chitosan microspheres* *(40–60 μm)*	w/o emulsion technique with glutaraldehyde as a cross-linker	67–90% / 7–45%	Human Breast cancer	[130]
*PG loaded biodegradable (PLGA)-based microparticles* *(5–50 μm)*	single emulsion solvent evaporation technique with (PVA) as an emulsifier	-	Human Breast cancer	[131]
*PG-conjugated AgNPs* *(9.98 nm)*	a rapid one-step method based on the amphoteric properties of silver oxide in alkaline solutions	-	Human Liver cancer	[132]
*PG- conjugated biosynthesized gold nanoparticles (AuNPs)* *(51–60 nm)*	A rapid method in presence of gold (III) chloride tri-hydrate, conjugated with LHRH peptide and addition of PG by physisorption	-	Human Breast cancer	[133]
*Dendrigraft poly-L-lysines PG loaded nanoparticles (DGL/CSA-PNPs)* *(396.10 nm)*	Synthetic placental chondroitin sulfate (CSA)-binding peptide (plCSA-BP) used to modify dendrigraft poly-L-lysines (DGL)	81–89% / 38–40%	Choriocarcinoma	[134]
*β-Cyclodextrin grafted magnetic nanoparticles and carboxymethyl chitosan-coated magnetic nanoparticles* *(38.2–121.1 nm)*	Two-step synthesis by co-precipitation of ferrous and ferric salts in a carboxymethylated β-Cyclodextrin. Single-step synthesis of chitosan-coated Fe_3_O_4_. PG loaded via inclusion complexation and adsorption	81–92% / 56–59 mg per 100 mg MNPs	Human Breast cancer/Human Liver cancer	[136]
*PG nanomicelles* *(223.8/217.1 nm)*	Microbial fermentation in presence of Tween 80	-	*Antimicrobial against* *Staphylococcus aureus* and *Escherichia coli*	[19,137]
*κ-carrageenan and maltodextrin PG loaded microparticles* *(0.5–5 µm)*	Spray-dried technique with κ-carrageenan and maltodextrin as encapsulation agents	-	*Enhanced the natural pigment’s properties as a colorant*	[138]
*PG loaded P(NIPA)-based hydrogels, P(NIPA-co-AM), and P(NIPA-co-BMA)*	P(NIPA)-based hydrogels were prepared by free-radical polymerization	-	*Human Breast cancer*	[141,142]
*Hybrid composite nanofibers* *(1–1.4 μm)*	Developed by electrospinning with PLGA, gelatin, pluronic F127, and PG	54%/ 3.60 mg mL^−1^ of PLGA/Ge-F127/PG	*Human Breast cancer*	[145]
*PDMS implantable packages* *(2–4 mm)*	PDMS packages containing PG-storing hydrogel (PNIPA and co-monomers of AM and BMA)	-	*Human Breast cancer*	[146,147]

* EE (Encapsulation efficiency) / DL (Drug loading)

### 7.1 Micro- and nano-particles

The encapsulation of different drugs into nano and microparticles represents a successful improvement for the targeted delivery of chemotherapeutic agents to enhance the therapeutic activity and reduced its side effects. In one study, PG-loaded chitosan microspheres had been produced with the water-in-oil (w/o) emulsion technique, using glutaraldehyde as a cross-linker. They presented an average size of 40–60 μm and an encapsulation efficiency (EE, %) between 66.7 ± 3% and 90 ± 4%. The action of the drug release was tested on viable breast cancer cells (MDA-MB-231 cells), and a notable decrease in the cell viability was observed after 24 h with the PG treatment, unlike the control sample in which cell proliferation was observed [130].

In another approach for cancer delivery, biodegradable poly(D,L-lactide-coglycolide) (PLGA)-based microparticles containing PG had been formulated using a single emulsion solvent evaporation technique with poly-vinyl alcohol (PVA) as an emulsifier. Also, particles loaded with paclitaxel (PTX, anticancer drug) were produced as a control. PG-loaded microspheres presented particle sizes between 5 and 50 μm and relatively high and similar drug loading efficiency and EE to the PTX-loaded PLGA microspheres, suitable for controlled and localized drug delivery in cancer treatment. Their cytotoxicity was tested against breast cancer cells MDA-MB- 231 and the results showed a similar effect compared to PTX since PG-loaded microspheres induced apoptosis by stopping the progression of the cells during a mitotic stage of the cell cycle [131].

There is a wide variety of metal nanoparticles for biomedical applications. PG-conjugated AgNPs (PG-AgNPs) were synthesized by a rapid one-step method based on the amphoteric properties of silver oxide in alkaline solutions. These highly stable and spherical nanoparticles presented a mean diameter of 9.98 nm and were tested against a human liver cancer cell line (HepG2) showing an IC_50_ value of 29.85 μg mL^−1^ while free PG presented an IC_50_ of 44.83 μg mL^−1^ [132]. An interesting approach for obtaining gold nanoparticles was carried out from *Nauclea latifolia* leaf extracts in a rapid method (less than 30 s) in presence of gold(III) chloride tri-hydrate, once the AuNPs were obtained, they were conjugated with LHRH peptide and then followed by the aggregation of PG utilizing physisorption. That AuNP-LHRH/PG nanoparticles presented an average size between 51 and 60 nm and a diversity of shapes, such as prismatic, octagonal, heptagonal, and hexagonal shapes. The adhesion of the nanoparticles to MDA-MB-231 cells and normal breast cells was studied using Atomic Force Microscopy (AFM), which showed a five times bigger force of adhesion for the LHRH conjugated nanoparticles to MDA-MB-231 cells than AuNPs and normal breast cells. A similar result was observed for the AuNP-LHRH/PG, which could be related to the overexpression of LHRH receptors on the surfaces of MDA-MB-231 breast cancer cells. Although cytotoxicity assays remain undone, this system showed great potential for selective and specific targeting and treatment of breast cancer [133].

A similar strategy for targeting delivery was applied using synthetic placental chondroitin sulfate (CSA)-binding peptide (plCSA-BP) derived from malarial protein VAR2CSA to modify dendrigraft poly-L-lysines (DGL). This resulted in PG-loaded nanoparticles (DGL/CSA-PNPs) with an average diameter of 396.10 ± 13.27 nm and with a negatively charged surface, the zeta potential of −6.94 ± 0.65 mV. The nanoparticles presented a loading capacity of 41.36 ± 0.87% and an encapsulation efficacy of 89.39 ± 1.83%. *In vitro* release studies revealed a burst-release within 12 h at both pH 5.3 and 7.4 showing this could be used as a delivery carrier. Their cytotoxic effect was evaluated on choriocarcinoma cells (JEG3 cells), a highly malignant neoplasm, showing a significantly higher anticancer activity than free PG *in vitro* and also in the JEG3 tumor model *in vivo*. Additionally, further studies indicated that apoptosis was triggered mainly by the mitochondria-cytochrome c pathway. These results suggested that this system had great potential for cancer-targeting and antitumor efficacy [134].

An interesting synergistic action against bacteria of PG conjugated with an iron-oxide-activated carbon composite ([Ac]F@Fe3O4− PG) was observed. The developed pigmented system resulted interesting for water purification purposes and was tested against antibiotic-resistant *E. coli* and *B. cereus*. The results showed a combination of cell membrane damage due to the surface charge neutralization by cationic [Ac]F@Fe3O4− PG and triggering apoptosis by the generation of reactive oxygen species. On *E. coli*, a complete bactericidal efficiency was observed at 4 mg mL^−1^ of [Ac]F@Fe3O4− PG, while 5 mg mL^−1^ was needed for *B. cereus*. Also, this matrix was demonstrated to be reusable and presented a long-term antibacterial activity [135].

In another work, two glucose-based smart tumor-targeted drug delivery systems coupled with an enzyme-sensitive strategy had been developed using magnetic nanoparticles (Fe3O4) grafted with carboxymethyl chitosan (CS) and β-cyclodextrin (β-CD) as carriers. β-CD and CS-MNPs were measured by DLS presenting diameters of 121.1 and 38.2 nm and an EE of 81% and 92%, respectively. *In vitro* release studies were made in the presence of alpha-amylase and chitosanase and the results showed that 58.1% and 44.6% of the pigment were released after 1 h of incubation. Also, their cytotoxic activity was studied on two cancer cell lines, MCF-7 and HepG2, and non-cancerous NIH/3T3 cells), resulting in a selective index (SI) of 7.03 on MCF-7 cells for CS-MNPs, markedly greater than β-CD-MNPs (SI 1.27) and free PG (SI 1.54) [136].

**Figure 4.**
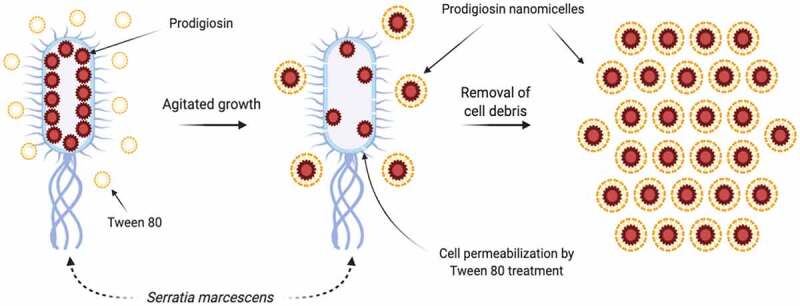
Schematic process of the prodigiosin nanomicelles production (modified from Gong et al.[18]).

The antimicrobial activity of this pigment was used to develop a dyeing process of cotton with PG nanomicelles. Those nanomicelles were obtained by microbial fermentation when Tween 80, a nonionic surfactant, was added to the culture media so the hydrophobic pigment was wrapped into the micelles of the surfactant under continuous oscillation (Figure 4). The system presented an average diameter of 223.8 nm and the dyed cotton showed excellent bacteriostatic rates, 99.2% and 85.5% against *S. aureus* and *E. coli*, respectively [19]. The same strategy was applied for a dying process for silk based on PG. The mean particle size of the PG nanomicelles was 217.1 nm with a polydispersity index of 0.199. The antibacterial activity was tested against *S. aureus* and *E. coli*, resulting in a greater antibacterial rate for *S. aureus* (93.2%) than the one for *E. coli* (25.1%). Also, the *in vitro* cytocompatibility of the dyed silk was studied on L929 cells and no toxic effects were seen compared to undyed silk after 1, 2, and 3 days of static incubation [137]. Other research exhibited the possibility of encapsulating PG into microparticles using the spray-drier technique with κ-carrageenan and maltodextrin as encapsulation agents. These smooth microparticles presented mean diameters ranging from 0.5 to 5 µm. Although cytotoxicity assays were not tested, the present system showed some interesting properties concerning the increase of PG solubility [138]. Hence, further research would be necessary and become an interesting field to be explored.

It was reported that PG-loaded halloysite-based nanoformulation affected the viability of malignant cells (human epithelial colorectal adenocarcinoma (Caco-2) and human colon carcinoma (HCT116) cells) and not the viability of nonmalignant cells (MSC, HSF). In the case of malignant cells, it was observed disorganization of the F-actin structure. The conclusion was that PG exhibited a selective cytotoxic and genotoxic activity in the nanoformulation [139].

In recent work, PG was loaded into two types of metal-organic framework nanocarriers: MIL-100(Fe) and MIL-100(Fe)/Folic Acid for targeted delivery against folic acid-positive breast cancer cells, MCF-7. A loading capacity of around 40% with 93–97% loading efficacy of both nanocarriers was found, as well as a PG controlled release. The selective index from 3.21 to 12.48 indicated that folic acid nanoparticles could improve the anticancer potential of PG. [140]

### 7.2 Hydrogels

There are other studies based on macroscopic systems with the same objective of improving PG delivery. Smart hydrogels had been developed based on poly(N-isopropyl acrylamide) due to the ability of these (P(NIPA))-based polymers to expel their liquid contents at human temperature (~37°C). P(NIPA)-based homo-polymer and P(NIPA)-based co-polymers with acrylamide (AM) and butyl methacrylate (BMA) had been studied, and the swelling due to the uptake of PG as compared to control environments: distilled water (DW), paclitaxel™ (PT) and bromophenol blue (BB). The P(NIPA)-based homopolymer hydrogels presented an average porosity of 0.34, while P(NIPA-co-AM) was between 0.53 and 0.67 and P(NIPA-co-BMA) 0.39 and 0.48. The drug loading efficacy could be regulated by temperature and 29°C seemed to be the better option, while drug elution could be controlled at 37°C, or above that temperature (41°C, 43°C, and 45°C). The diffusion coefficients of PG released from the hydrogels were between 4.97 and 9.29 × 10^−9^ m^2^ s^−1^ at 37°C. The results suggested this system could be used to control the release of drugs during localized chemotherapy, although further research remains needed, such as studying the effects on cancer cell lines, and biocompatibility studies [141]. With further analysis, the kinetics of drug release was studied, and a Fickian diffusion-controlled release was observed for the P(NIPA)-based homopolymer and P(NIPA)-co-AM (90:10 wt.%) at 37°C. Also, the *in vitro* effect of the drug-loaded hydrogels was studied on MDA MB 231 and MCF 10A breast cancer cell lines. The results indicated an exponential decrease in cell survival (%) as a function of incubation time with no significant differences in the composition of the hydrogels. Statistical differences in the metabolic activity were found comparing non-treated and treated cancer cells but also in the normal cells [142].

The demonstrated antimicrobial activity of the PG motivated the development of a column reactor containing cellulose membrane impregnated with the pigment for the elimination of pathogenic microorganisms, such as *B. cereus* and *E. coli* present in water with a similar efficiency of about 97.3% for both bacteria [143].

A similar strategy for food packaging was developed using bacterial cellulose (BC) membrane composites functionalized with chitosan and polyvinylalcohol (PVA) doped with PG. High antimicrobial activities of the bacteria cellulose composites containing PG against *P. aeruginosa* (PA25) and *S. aureus* (ATCC 6538) tested under ASTM E2180-07 standards. The authors suggested that BC membranes containing PG could be used in food packaging to avoid food spoilage of fresh foods [144].

### 7.3 Implantable devices

Different approaches were made to the design of a matrix or a device that can offer a suitable drug release for a localized region ensuring a better action of the treatment, avoiding side effects, and lowering the required dose.

**Figure 5.**
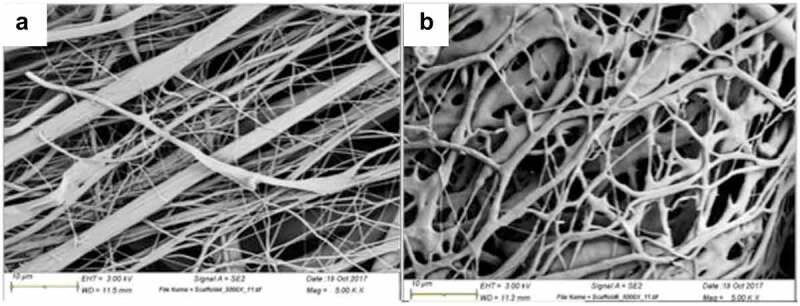
SEM micrograph showing the morphologies of (a) PLGA/Ge-PG electrospun scaffold and (b) PLGA/Ge-F127/PG scaffold (with permission from [117]).

Hybrid composite nanofibers were developed by electrospinning with poly(D,L-lactic-co-glycolic acid) (PLGA), gelatin, pluronic F127, and PG looking forward to an improvement in cell adhesion as well as a sustainable drug release profile (Figure 5).

The mean diameters obtained from SEM images were 1.0 ± 0.9 μm and 1.4 ± 1.3 μm for PLGA/Ge-PG and PLGA/Ge-F127/Ge respectively. The tensile stiffness of the nanofibers was studied by calculating Young’s moduli which resulted to be 1.446 ± 0.496 and 1.290 ± 0.617 kPa, while the ultimate tensile strengths were 0.440 ± 0.117 kPa and 0.185 ± 0.480 kPa for PLGA/Ge-PG and PLGA/Ge-F127/Ge, respectively. The last one presented an improved EE of 54%, due to the presence of the surfactant (Pluronic F127), while the EE for PLGA/Ge-PG was 38%. That was also reflected in the drug loading capacity where it went from 1.899 mg mL^−1^ of PLGA/Ge-PG to 3.60 mg mL^−1^ of PLGA/Ge-F127/PG. The study of *in vitro* drug release from this system showed an initial burst for a period of 1 h to be 26.000 ± 0.004 vol% and 16.000 ± 0.015 vol% for PLGA/Ge and PLGA/Ge-F127 nanofibers, respectively. The cumulative release of PG (for 3 days) was determined to be 82.0 ± 0.1% for PLGA/Ge and 49.7 ± 0.1% for PLGA/GeF127 nanofibers. Both nanofibers exhibit diffusion-controlled release by non-Fickian (zero-order) and quasi-Fickian diffusion in the initial and sustained release regimes, respectively. The *in vitro* effects of PG release was tested on two cancer cell lines, MCF-7 and MDA-MB-231. PLGA/Ge-PG nanofibers exhibited a significant cell viability reduction in comparison with control samples (Tissue Culture Plate and PLGA/Ge). Moreover, the electrospun nanofibers (without the drug) improved the viability and proliferation of cancer cells better than the TCP [145].

Another work proposed an implantable encapsulated structure that can deliver localized heating (hyperthermia) and controlled concentrations of PG composed of Poly-di-methyl-siloxane (PDMS) packages, which contain a drug-storing hydrogel produced with Poly(N-isopropyl acrylamide) (PNIPA) co-monomers of acrylamide (AM) and butyl-methacrylate (BMA). Dried PNIPA-based hydrogels were inserted into the PDMS capsules, and after sealing, the devices were saturated in PG solutions. The hydrogels presented an average diameter between 2 and 4 mm. The diffusion kinetics of PNIPA gels was studied at temperatures of 28°C to 48°C, fitting conditions for cancer treatment via hyperthermia and drug delivery. Drug release rates were dependent on time, while the drug diffusion rates were strongly influenced by temperature. Non-Fickian diffusion dominated the behavior of the hydrogels and presented a mesh size of about 5–100 nm [146].

Further work was done analyzing the effect on cell viability of these devices via clonogenic assay testing on the MDA-MB-231 breast cancer cell line, compared to the effect of the control drug PTX. Also, the action of the drugs was studied in a range of temperatures (37–45°C) simulating a hyperthermia treatment. Both drugs, PG and PTX, prevented colony formation and significantly reduced the cell viability on a drug release assay of 72 h. The combined effect of drug release and hyperthermia was studied obtaining a significant increase in cell death as both, drug dose and temperature, increased. Similar results were obtained for PG release compared to PTX release at 37–43°C, also relevant to hyperthermia-induced loss of cancer cell viability [147].

### 7.4 3D printing potential uses

**Figure 6.**
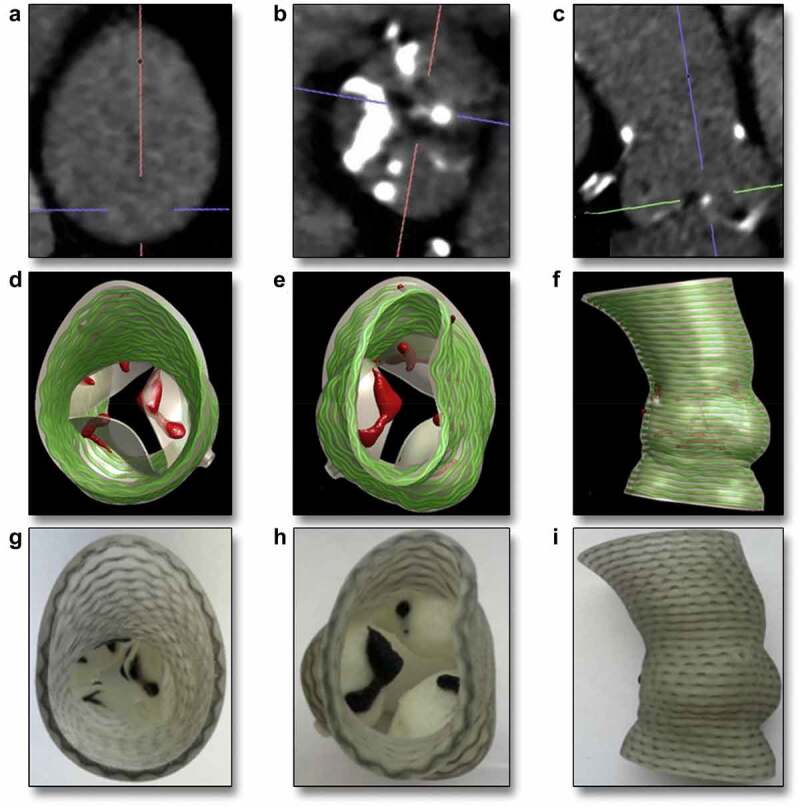
The potential application of 3D printing to design aortic root starting from the computed tomography cross-sectional view, followed by the 3D computational Model to finally obtain a 3D-Printed phantom (with permission from [121]).

In recent years, techniques based on three-dimensional (3D) printing have been gaining importance in the research field by developing numerous improvements, attracting the interest of industrial companies worldwide. The 3D printing industry is experiencing rapid growth, and it has excelled in the medical field. Recent advances in additive manufacturing allowed the development of different strategies with great potential. Hybrid scaffolding materials to achieve tunable properties of scaffolds and biodegradable polymers, such as PLA and PLGA have been used commonly as base materials alone or mixed with additives for improvement of some of their properties (Figure 6) [148]. Another strategy is the design of special microstructures to achieve the convertibility of scaffolds. The structure of the scaffold can be modified using auxetic metamaterials, which are composed of repeated microstructures and allow the volume to change providing an effective way to control the local cell density. Integration of sensors to achieve built-in process-monitoring capability, with direct-write technologies, such as inkjet printing or aerosol jet printing, opens the possibility to create smart scaffolds. The aerosol jet printing, for example, can print on non-planar substrates providing the opportunity of integrating sensors into bio-printed scaffolds [149].

3D printing has interesting advances in the matter of drug delivery allowing the creation of specifically tailored dosage forms according to each patient and their condition. This is possible using biodegradable polymers, which can be drug-loaded by incorporating the drug into the filament or soaking it in a drug solution [150].

A 3D printing technique, binder jetting technology (BJ), is the base for the development of the first 3D printed medicine approved by the US FDA, Spiritam® (levetiracetam), which consists of a highly porous tablet developed by the Zipdose® technology, with a rapid dispersion upon contact of liquids. Semi-solid extrusion 3D printing is a technique where the starting materials, which present a semi-solid consistency, are extruded via a syringe-based tool-head nozzle to create the 3D object, and researchers have made interesting progress in the pharmaceutical field as high drug loading or multiple drugs formulations have been manufactured [151].

As seen through bibliographic search, 3D printing techniques offers highly varied potential uses, but there is a need for further research on drug delivery and also no PG-related studies were found which results in a great opportunity to explore.

## 8. Future potential

It has been demonstrated that PG exerts a wide spectrum of activities. This fascinating fact is a true promise to continue discovering other new activities of this versatile molecule. The linear tripyrrole structure of PG was used as a platform for the synthesis of a new family of pyrrolic compounds tailored by the addition of new functional groups that increase and provide high specificity to diverse biological activities. In addition, PG could be complexed with different macromolecules (*i.e*., amino acids, proteins) to allow the creation of new pigment complexes with novel properties [152,153].

Regarding molecular modeling, docking appears as a very interesting method to predict and evaluate the *in-silico* biological activity of PG for particular diseases. The preferred orientation of PG molecule to face other molecules or active sites become a very interesting tool to study particular bounds and the potential formation of stable complexes [154].

Also, PG molecules could offer diverse coordinating properties toward different metals, even in the presence of multiple oxidation states. In this sense, the pyrrolyldipyrrin motifs of PG showed a very interesting ligand behavior with the ability to form stable ion-PG complexes [155]. It was described that PG-metal complexes in the presence of an oxidant environment could cleave double-stranded DNA due to the formation of a ligand-based π-radical cation. This point opens a new gate to explore the antiviral activity of PG, which is still unexplored, and this is vital considering the emergence of new viral pathogens, most of them showing a DNA or RNA-based structure.

Finally, more intense studies in the field of medicinal chemistry are required to explore more potentialities for PG and the reasons for its diverse biological activity profiles. Those studies should be carried out carefully linked to the development of new drug delivery systems, which could facilitate the vehiculation of PG and enhance its activity in specific biological targets.

## Final remarks

PG emerged as an interesting biotechnological molecule mainly produced by *Serratia marcescens*. Also, PG was used as a platform to bring a family of molecules with diverse biological activities obtained by chemical synthesis. Modification and tailoring of the environmental growth conditions of the strain result in different PG yields. The unique pyrrolyl pyrromethane structures of the molecule provide a wide variety of biological activities working as antimicrobial, antifungal, anticancer, antimalarial, or antiviral agents that could have a clear impact on the improvement of world health. The production of PG could be carried out with sustainable material raw and, even with secondary metabolites that come from industrial wastes. However, its action is limited due to the high hydrophobicity that negatively impacts the bioavailability and low solubility in biological environments. In this sense, the vehiculation of PG becomes a very interesting tool to be explored and to create new molecular delivery technologies. The development of micro and nanoparticles, hydrogels, or implantable devices for local and targetable drug delivery appears as promising alternatives. The use of PG associated with 3D printing technologies results in a non-explored tool to design new systems that could potentiate the PG activities. It was clear from all these facts that more *in vivo* experiments should be necessary to validate the use of PG in the treatment of different pathologies, including new diseases associated with the appearance of MDR microorganisms. Meanwhile, several phases I/II clinical studies with several cancer patients have occurred with a PG derivative called obatoclax, and its potentiality is still under study whether PG or nanostructures PG is an efficient treatment for human cancer patients or not, but this is a tremendous step to human therapy with PG.

However, many challenges are still around for the PG molecule to be available in the market. It is imperative the increase genetic knowledge, regulatory aspects, and metabolic network of the main PG producers reported in the literature, with a systematic and validated approach. Also, the search for hyper-producing stable strains and novel vectors of PG expression can open a new area for the development of novel PG-derivative able to target specific pathologies.

The development of laboratory cultivation techniques that allowed to be scaled up using cheap substrates and, associated with the development of a new purification process and protocols that allows establishing proper PG standards for regulatory applications and potential therapeutic uses.

## Conclusions

The present review exposed that the production of PG could be considered a new platform for the rational design of biotechnological products developed by microbial fermentation. The wide spectrum of biological activities described for PG makes this molecule an attractive candidate to establish new alternatives to overpass the multidrug resistance in different diseases, control the dissemination of microorganisms and avoid possible new pandemics. The high hydrophobicity of the molecule is not a limitation for its application in the physiological environment since many drugs in the market share similar limitations, and in addition novel systems for drug delivery systems have been proposed to increase its bioavailability.
